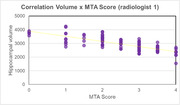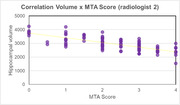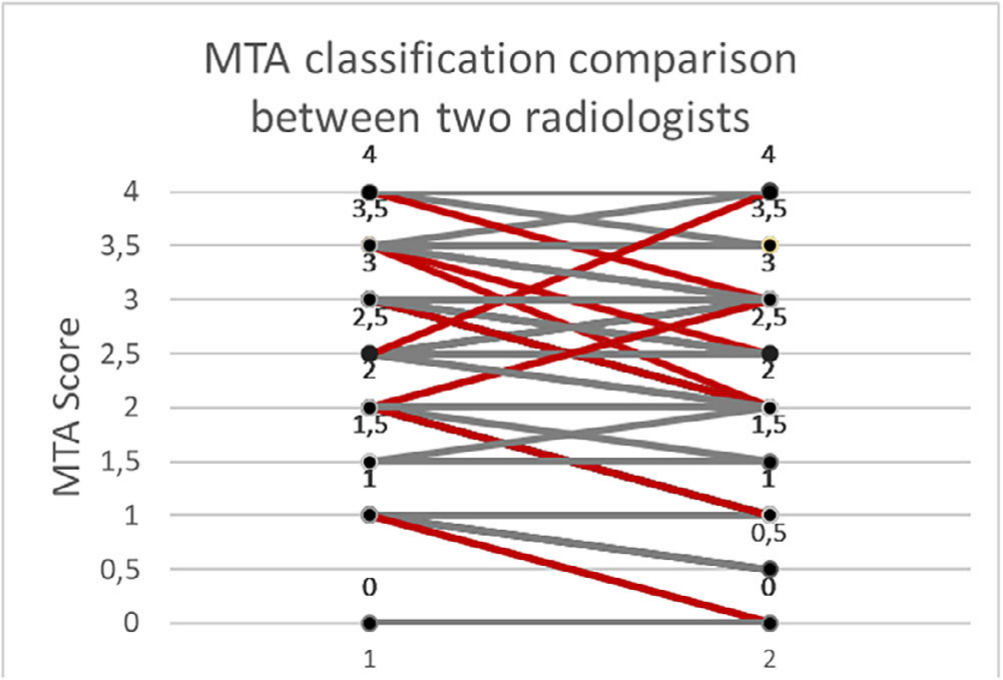# Association between hippocampal volume and visual classification of hippocampal atrophy: an MRI study

**DOI:** 10.1002/alz.091516

**Published:** 2025-01-09

**Authors:** Fabricio Nery Garrafiel, Vitor Verlindo Vidaletti, Ricardo Benardi Soder, Ricardo Pessini Paganin, Maria Rosa Alves da Silva, Cristiano Schaffer Aguzzoli, Lucas Porcello Schilling

**Affiliations:** ^1^ Pontifical Catholic University of Rio Grande do Sul, PORTO ALEGRE, RIO GRANDE DO SUL Brazil; ^2^ Brain Institute of Rio Grande do Sul, Porto Alegre, RS Brazil; ^3^ Brain Institute of Rio Grande do Sul, PUCRS, Porto Alegre, RS Brazil

## Abstract

**Background:**

Hippocampus has a key role in the memory and cognitive functions and its volume tends to decrease during aging and is severely affected in neurodegenerative disorders such as Alzheimer’s disease (AD). The hippocampal atrophy can be assessed by quantitative volumetry or classified by the Mesial Temporal Atrophy Score (MTA). Here, we perform a comparison between the values of automatic hippocampal volumetry and the MTA score, to analyze possible variations through a correlation curve and their impacts on quantitative and qualitative measures.

**Method:**

We analyzed 100 cranial Magnetic Resonance Imaging (MRI) images, T1‐weighted from the Alzheimer's Disease Neuroimaging Initiative (ADNI). The hippocampal volume was obtained by Freesurfer analysis. All images were classified using MTA score by two experienced neuroradiologists. In the statistical analysis, a correlation curve was created, and the Pearson correlation coefficient was obtained between the volumetry and the degree of atrophy and between the interpretations of both radiologists.

**Result:**

The hippocampus volumetric mean size was 2963.83 mm³ (standard deviation 495.16 mm³). As expected, the data exhibited a consistent inverse relationship between hippocampal volume and the MTA score (Figure 1A, B). The Pearson correlation showed a negative correlation between MTA score and hippocampal volume of ‐0.75 for one radiologist and ‐0.76 for the second radiologist and 0.89 between radiologists. However, we observed a variation ≥ 1 predominantly in higher MTA scores in 17 individuals when comparing the MTA scores between radiologists (Figure 2).

**Conclusion:**

The results showed an inverse correlation between MTA score and hippocampal volume, but also indicates discrepancies in visual interpretation of data. Our findings highlight that in some situations the MTA classification has an important mismatch compared to volumetry, and future analysis could clarify in which situations MTA is less accurate and should be complementary to other analysis.